# Comparative study of milk microbiota and metabolome in long-lived dairy cows with different persistent production capacities

**DOI:** 10.3389/fmicb.2025.1725031

**Published:** 2026-01-06

**Authors:** Jianhao Yang, Naihan Yuan, Tengfei Guo, Yanfei Feng, Shanshan Guo, Dong Zhou, Pengfei Lin, Aihua Wang, Yaping Jin

**Affiliations:** 1 College of Veterinary Medicine, Northwest A&F University, Yangling, China; 2 Key Laboratory of Animal Biotechnology, Ministry of Agriculture and Rural Affairs, Northwest A&F University, Yangling, China

**Keywords:** dairy cows, longevity, milk metabolome, milk microbiome, sustained productivity

## Abstract

Improving dairy cow lifespan is essential for sustainable livestock production. The milk microbiome and metabolome are closely associated with mammary gland health and influence the persistent productivity of dairy cows. However, the characteristics of the milk microbiome and metabolome underlying persistent productivity remain unknown. In this study, 16S rRNA sequencing and untargeted metabolomics were applied to evaluate the milk microbiome and milk metabolome composition of long-lived, high-yielding cows (LH) and long-lived, low-yielding cows (LL). The results showed that no significant differences were observed in the *α*- and *β*-diversity of milk microbiota between the two groups (*p* > 0.05). However, the community assembly processes differed significantly. The LH group exhibited significantly higher levels of homogeneous selection, drift (and others) (*p* < 0.05). In contrast, dispersal limitation, homogeneous dispersal, and heterogeneous selection were significantly lower (*p* < 0.05). In addition, in the milk of LH cows, *UCG-005*, *Prevotellaceae UCG-003*, *Ruminococcus*, *unclassified f Oscillospiraceae*, *norank f Fodinicurvataceae*, and *unclassified f Ruminococcaceae* were significantly enriched (LDA > 2, *p* < 0.05). The bacterial functions of protein digestion and absorption and N-glycan biosynthesis were significantly enriched in the LH group, while thyroid hormone synthesis and pathogenic *Escherichia coli* infection were significantly enriched in the LL group (LDA > 2, *p* < 0.05). Additionally, the milk of LH cows exhibited elevated levels of omega-3 polyunsaturated fatty acids (PUFAs), including PE (20:5/0:0), LPC (20:5 (5Z, 8Z, 11Z, 14Z, 17Z)/0:0), LPE (0:0/20:5 (5Z, 8Z, 11Z, 14Z, 17Z)), and PE (22:5/0:0) (LDA > 2, *p* < 0.05). Milk PE (18:3/0:0) showed a significant positive correlation with milk *Prevotellaceae UCG-003* and *UCG-005* (|*r*| > 0.50, *p* < 0.05). These bacterial genera were significantly negatively correlated with the predicted microbial function pathogenic *E. coli* infection (|*r*| > 0.50, *p* < 0.05). The accumulation of omega-3 PUFAs in milk may help maintain the homeostasis of mammary microbial environment and promote mammary health. These results provide novel insights into the microbial and metabolic signatures underlying persistent productivity, offering potential targets for nutritional and microbial interventions to enhance dairy cow longevity.

## 1 Introduction

Global dairy production is projected to increase by approximately 1.5% annually over the next decade (OECD, Food, and Nations, A.O.o.t.U, 2023). However, the sustainability of global food production and consumption remains suboptimal (Willett et al., 2019). Consequently, transitioning dairy production toward greater environmental sustainability and social acceptability has become a major priority (Adamie et al., 2023). The lifespan of dairy cows represents the length of their productive life within a herd. Extending this lifespan is associated with improved animal welfare, higher societal acceptance (Schuster et al., 2020), enhanced farm profitability (Owusu-Sekyere et al., 2023), and greater environmental sustainability in dairy production (Grandl et al., 2019). The overall lifespan of a dairy cow herd is strongly affected by involuntary culling, with up to 80% of such culling events related to health issues (Hare et al., 2006). During the early to mid-lactation period, the main reasons for culling are metabolic diseases and mammary gland infections (Probo et al., 2018). Therefore, the health of the mammary gland directly influences the productive lifespan of dairy cows (Pinedo et al., 2010).

The structure and composition of milk microbiota reflect mammary gland health. Dysbiosis of the commensal microbiota contributes to the development of mastitis (Zhao et al., 2022), while transplantation of milk microbiota from cows with clinical mastitis into mice can induce mastitis (Hoque et al., 2022). Mastitis in dairy cows decreases the abundance of probiotics such as *Lactobacillus* and *Bifidobacterium* in milk, and commensal bacteria, including *Dietzia*, *Aeromicrobium*, *Alistipes*, and *Sphingobacterium*, are also reduced (Qiao et al., 2015; Wang et al., 2020). The commensal bacteria may stabilize the milk microbiota by inhibiting pathogens through the production of organic acids and bacteriocins (Espeche et al., 2012; Nagahata et al., 2020). The milk microbiota also reflects the stability of the gut microbiota. Cows with mastitis exhibit rumen and fecal dysbiosis (Wang et al., 2021; Wang et al., 2022a). After gut microbiota dysbiosis, opportunistic pathogens such as *Escherichia* and *Shigella* can translocate via the gut-mammary axis, thereby promoting mastitis (Zhao et al., 2022).

The metabolic profiles of raw milk from dairy cows vary across various lactation stages. l-glutamate and l-tyrosine levels are higher during peak lactation, whereas l-phenylalanine, dulcitol, and 3-phenylpropyl acetate are elevated during mid-lactation (Wang et al., 2024). The metabolite composition of raw milk also differs significantly among species. For example, the levels of iminostilbene and osteopontin in cow milk are substantially higher than those in buffalo and yak milk (Li et al., 2024). Milk metabolites not only reflect nutritional value but also indicate mammary gland health. Pathogen-induced mastitis significantly alters the milk metabolite profile, directly reducing milk quality. In milk from cows with clinical mastitis, concentrations of glucose, D-glycerol-1-phosphate, l-carnitine, 4-hydroxyphenyllactate, citrate, and hippurate were reduced, whereas oligopeptides, such as Leu-Ala, Phe-Pro-Ile, Asn-Arg-Ala-Ile, and Val-Phe-Val-Tyr, were elevated (Xi et al., 2017). Pathogen-mediated metabolic alterations can further exacerbate mastitis. During mastitis, reduced xanthine levels induced by elevated enterohemorrhagic *E. coli* (EHEC) counts in milk suppress mammary gland stem cell populations, thereby impairing tissue repair (Choudhary and Capuco, 2012; Crane et al., 2013; Wang et al., 2020). In addition, milk metabolomics can reflect the energy balance of dairy cows. In cows with a negative energy balance, milk levels of citrate, cis-aconitate, creatinine, and glycine were elevated, while choline, ethanolamine, fucose, and *N*-acetylneuraminic acid were reduced (Xu et al., 2020).

Given the important role of the milk microbiome and metabolome in dairy cow health and productivity, we hypothesized that long-lived cows with sustained production capacity possess distinct milk microbial and metabolic characteristics. Therefore, the objective of this study was to characterize and compare the milk microbiome and metabolome of long-lived cows with different production capacities and to evaluate their potential roles in sustaining milk production.

## 2 Materials and methods

### 2.1 Ethics approval statement

This experiment was conducted at the Animal Research and Technology Center of Northwest A&F University, and it was performed in accordance with the recommended guidelines from the Administration of Affair Concerning Experimental Animals (Ministry of Science and Technology, China, revised 2004). The protocol was approved by the Institutional Animal Care and Use Committee at Northwest A&F University (Ethical Approval number: DY2022093).

### 2.2 Animal, study design, and sample collection

A total of 219 healthy, long-lived Holstein-Friesian dairy cows were all born and raised on the same commercial dairy farm in Lingwu, Ningxia, China (37°49′N, 106°21′E), and with five to seven parities, were recruited (Group 219, parity 5.42 ± 0.77, days in milk (DIM) 148.19 ± 41.09, and BCS 2.5–4.0). All cows were maintained under comparable environmental and management conditions, housed in free-stall barns equipped with bedding and an exercise yard, with ad libitum access to drinking water. No substantive environmental differences existed among animals. Furthermore, once cows were identified as having longevity potential, they were relocated to a designated barn for long-term rearing, where environmental factors—including housing conditions, diet, and caretakers—were kept highly consistent across individuals. The dairy cattle population consists of long-lived cows with a historical average 305-day milk yield (305-day MY) greater than 10,000 kg/parity. The selection criteria included no history of antibiotic use in the past 3 months, no lifetime history of reproductive disorders, similar body condition scores (BCS), consistent feeding and management practices, and comparable parity and days in milk (DIM). A total of 54 cows were selected, with an average parity of 6.13 ± 0.14, DIM of 130.06 ± 4.74, and BCS ranging from 2.5 to 3.5. Based on historical average 305-day MY, eight long-lived high-yielding cows (LH) and eight long-lived low-yielding cows (LL), with comparable parities (5.94 ± 0.19), DIM (130.88 ± 1.95), and BCS (3.33 ± 0.12), were selected. An experienced veterinarian conducted clinical examinations and confirmed that all cows were in a healthy condition. All cows were given the same diet: 55% concentrate and 45% roughage on a dry matter (DM) basis (Supplementary Table S1). The cows were milked daily at 06:00, 14:00, and 22:00, and fed at 06:30, 14:30, and 22:30. One week before the experiment, experienced farm staff assessed each cow’s BCS and scored them on a scale of 1–5 in 0.25 increments (Edmonson et al., 1989). Over a 7-day period, the MY of each cow was recorded, along with the initial and residual feed weights, to calculate dry matter intake (DMI) and feed efficiency (FE). On the final day, prior to morning feeding, rumen fluid samples were collected via an oral gastric tube, with the first 50 mL discarded to minimize salivary contamination. The samples were filtered through four layers of sterile gauze and stored for subsequent volatile fatty acid (VFA) analysis. Blood samples were collected from the tail vein before morning feeding and centrifuged at 3,500×*g* for 15 min at 4 °C in tubes without anticoagulant to separate serum for serum biomarkers analysis. On the same day, milk samples were collected in a 4:3:3 ratio based on the three milking times for subsequent milk composition, microbiome, and metabolomics analyses. Milk composition was determined using a Foss-4000 spectrophotometer (Foss Electric A/S, Hillerød, Denmark). For the microbiome analysis, a sufficient volume of milk samples (50 mL) was collected and centrifuged at 12,000×*g* for 15 min to remove the fat and whey layers, with the bacterial pellet retained for subsequent analysis.

### 2.3 Measurement of rumenal volatile fatty acid (VFA) concentration

Volatile fatty acids (VFAs) were determined by gas chromatography (Agilent 7820A, Santa Clara, CA, USA) equipped with a capillary column (AE-FFAP, 30 m × 0.25 mm × 0.33 μm; ATECH Technologies Co., Lanzhou, China), as previously described (Yang et al., 2025). Rumen fluid samples were treated with metaphosphoric acid, then centrifuged, and the resulting supernatant was mixed with caprylic acid, which served as the internal standard. After filtration through a 0.45 μm membrane, samples were analyzed under the following chromatographic conditions: injector and detector temperatures of 200 °C and 250 °C, respectively, and a temperature program from 45 to 150 °C at 20 °C/min, followed by a 5-min hold. Individual VFAs (acetate, propionate, isobutyrate, butyrate, isovalerate, valerate, 4-methylvalerate, and caproate) were quantified using calibration curves (25–800 μM) with 2-ethylbutyrate as the internal standard. Total VFA concentration was calculated as the sum of all individual VFA.

### 2.4 Measurement of serum biomarker concentrations

The serum concentrations of various biomarkers, including total protein (TP), albumin (ALB), globulin (GLB), alanine aminotransferase (ALT), aspartate aminotransferase (AST), alkaline phosphatase (ALP), total bile acids (TBA), gamma-glutamyl transferase (*γ*-GGT), blood urea nitrogen (BUN), creatinine (CRE), lactate dehydrogenase (LDH), glucose (GLU), total cholesterol (TC), triglycerides (TG), high-density lipoprotein cholesterol (HDL-C), low-density lipoprotein cholesterol (LDL-C), immunoglobulin G (IgG), immunoglobulin A (IgA), superoxide dismutase (SOD), glutathione peroxidase (GSH-PX), total antioxidant capacity (T-AOC), total glutathione (T-GSH), and catalase (CAT), were measured by AutoAnalyzer KHB-1280 (Shanghai Kehua Bio-Engineering Co., Ltd., Shanghai, China) and commercial assay kits from Beijing Jinhai Keyu Biotechnology Development Co., Ltd. (Beijing, China). The concentrations of non-esterified fatty acids (NEFA), beta-hydroxybutyrate (BHB), haptoglobin (HPT), serum amyloid A (SAA), prolactin (PRL), insulin (INS), glucagon (GCG), insulin-like growth factor 1 (IGF-1), growth hormone (GH), cortisol (COR), triiodothyronine (T3), and thyroxine (T4) were determined using enzyme-linked immunosorbent assay (ELISA) kits from Shanghai Keshun Bioengineering Institute (Shanghai, China). Levels of tumor necrosis factor-alpha (TNF-*α*), interleukin-1 beta (IL-1β), interleukin-6 (IL-6), interleukin-10 (IL-10), and malondialdehyde (MDA) were measured using ELISA kits from Nanjing Jiancheng Bioengineering Institute (Nanjing, China). Protein carbonyl (PC) levels were determined using ELISA kits from Solarbio Science & Technology Co., Ltd. (Beijing, China). All assays were performed according to the manufacturers’ protocols, and each measurement was conducted in triplicate, with mean values calculated for analysis.

### 2.5 Milk DNA extraction and 16S rRNA gene sequencing

Total genomic DNA was extracted from milk samples using the Norgen Milk Bacterial DNA Isolation Kit (Norgen Biotek, Thorold, ON, Canada), and DNA quality was assessed by 1% agarose gel electrophoresis, with concentration and purity measured on a NanoDrop 2000 spectrophotometer (Thermo Scientific, USA). The V3–V4 region of the 16S rRNA gene was amplified using primers 338F (5′-ACTCCTACGGGAGGCAGCAG-3′) and 806R (5′-GGACTACHVGGGTWTCTAAT-3′) carrying unique barcodes (Liu et al., 2016). PCR amplification was performed in a 20 μL reaction mixture containing buffer, dNTPs, primers, polymerase, and 10 ng template DNA. The thermal cycling program consisted of an initial denaturation at 95 °C for 3 min, followed by 27 cycles of 95 °C for 30 s, 55 °C for 30 s, and 72 °C for 30 s, with a final extension at 72 °C for 10 min (ABI GeneAmp® 9,700). PCR products were separated on 2% agarose gels, purified using a PCR clean-up kit (YuHua, Shanghai, China), quantified with a Qubit 4.0 fluorometer (Thermo Fisher Scientific, USA), and pooled in equimolar amounts. Sequencing was performed on an Illumina NextSeq 2000 platform (Illumina, San Diego, USA) according to the standard protocols of Majorbio Bio-Pharm Technology Co., Ltd. (Shanghai, China). Raw paired-end reads were processed for QC using fastp (Chen et al., 2018) (https://github.com/OpenGene/fastp, version 0.19.6), and merged using FLASH (Magoč and Salzberg, 2011) (http://www.cbcb.umd.edu/software/flash, v1.2.11).

After QC and merging, sequences were de-noised with the DADA2 plugin (Callahan et al., 2016) in QIIME2 (Bolyen et al., 2019), generating amplicon sequence variants (ASVs). Chloroplast and mitochondrial sequences were removed. To standardize sequencing depth, all samples were rarefied to 20,000 reads, with an average Good’s coverage of 99.09% (Supplementary Table S3). The rarefied data were then converted into relative abundances. Taxonomic assignment was performed using BLAST in QIIME2 against the SILVA 16S rRNA database (v138) (Quast et al., 2013). Functional prediction was carried out with Phylogenetic Investigation of Communities by Reconstruction of Unobserved States (PICRUSt2) (v2.2.0) with reference to the KEGG pathway database (Douglas et al., 2020). Raw sequencing data have been deposited in NCBI under BioProject accession number PRJNA1307952.

### 2.6 Untargeted metabolomics analysis of milk

Milk samples (100 μL) were extracted with 400 μL of acetonitrile:methanol (1:1, v/v) containing l-2-chlorophenylalanine (0.02 mg/mL) as the internal standard. After vortexing, the samples underwent low-temperature ultrasonication (5 °C, 40 kHz, 30 min), incubation at −20 °C (30 min), and centrifugation (13,000 g, 15 min, 4 °C). The resulting supernatants were dried under nitrogen and reconstituted in 100 μL of acetonitrile:water (1,1, v/v). The reconstituted solution was further ultrasonicated for 5 min and centrifuged (13,000 g, 10 min, 4 °C), and the supernatants were transferred to vials for analysis. QC samples were prepared by pooling equal aliquots of all extracts and injected every 5–10 runs to monitor reproducibility. Metabolomic profiling was performed on a UHPLC-Orbitrap Exploris 240 system (Thermo Scientific, USA). A 3 μL aliquot of each sample was separated on an HSS T3 column (100 × 2.1 mm, 1.8 μm) with mobile phase A (0.1% formic acid in water/acetonitrile, 95, 5, v/v) and mobile phase B (0.1% formic acid in acetonitrile, isopropanol, water, 47.5, 47.5, 5, v/v/v). The flow rate was 0.40 mL/min, and the column temperature was maintained at 40 °C. MS detection was carried out in both positive and negative ion modes (m/z 70–1,050), with spray voltages of +3,500 V/−3,000 V, sheath and auxiliary gas setting at 50/13 arb, an ion source temperature 450 °C, and a stepped collision energy of 20–40–60 V.

Raw LC–MS data were processed in Progenesis QI software (Waters Corporation, Milford, USA) for baseline correction, peak detection, integration, retention time alignment, and data normalization, generating a data matrix containing retention time, m/z, and peak intensity. MS and MS/MS spectra were matched against the Human Metabolome Database (HMDB)^1^, KEGG^2^, METLIN^3^, and the Majorbio database to obtain key information such as m/z, adducts, formula, and fragmentation score. Data preprocessing included the 80% rule for missing value filtering, imputation with minimum values, total sum normalization, removal of variables with RSD > 30% in QC samples, and log10 transformation. Multivariate analysis was conducted using OPLS-DA with 7-fold cross-validation in the R package ropls (v1.6.2). Differential metabolites were identified as those with VIP > 1 and *p* < 0.05 (Wilcoxon rank-sum test). Functional annotation of metabolites was performed using the KEGG database^4^, and pathway enrichment was analyzed in MetaboAnalyst 5.0 (Pang et al., 2021), using *Bos taurus* as the reference library, and weighted topological analysis was applied to calculate pathway significance and impact.

### 2.7 Statistical analysis

Physiological parameters, serum biomarkers, and rumen VFA concentrations were analyzed using SAS software (version 9.4, SAS Institute Inc., Cary, NC, USA). A mixed procedure was used as follows:


$$Y_{\mathrm{ijk}}=\mu +G_{i}+KC+e_{\mathrm{ijk}}$$

where $Y_{\mathrm{ijk}}$ is the dependent variable, μ is the overall mean, $G_{i}$ is the effect of the $\;i^{\mathrm{th}}\;$ group, Cis the vector of the fixed covariates, consisting of parity and DIM. $e_{\mathrm{ijk}}$ is the residual error. Model parameters were estimated using the default REML method and default denominator degrees of freedom settings in PROC MIXED. The treatment group was specified as a categorical variable using the CLASS statement. Least squares means were used to compare differences between groups. The Wilcoxon rank-sum test was applied to identify differential *α*-diversity and bacterial functional profiels (*p* < 0.05). *β*-diversity was assessed by principal coordinate analysis (PCoA) based on Bray–Curtis distances. Linear discriminant analysis effect size (LEfSe) was used to identify differential bacterial genera (LDA > 2, *p* < 0.05). A phylogenetic tree of milk bacterial genera was constructed using IQ-TREE (v3.0.1) with the maximum likelihood method. The iCAMP package (v1.5.12) was employed to evaluate ecological assembly processes in the milk microbiota (Ning et al., 2020). Spearman’s rank correlation coefficients were calculated among differential physiological parameters, serum lipids, milk bacterial genera, and milk metabolites, with significance defined as |*r*| > 0.5 and *p* < 0.05. Correlation networks and heatmaps were visualized using the ComplexHeatmap package (v2.21.0) and Cytoscape (v3.9).

## 3 Results

### 3.1 Physiological differences between LH and LL cows

Milk composition did not differ significantly between the two groups (*p* > 0.05; Table 1). In contrast, the LH cows exhibited significantly higher MY, milk fat yield (MFY), milk protein yield (MPY), energy-corrected milk (ECM), fat-corrected milk (FCM), feed efficiency (FE), 305-day MY (*p* < 0.001), and historical average 305-day MY (*p* < 0.01; Table 1). Regarding rumen fermentation parameters, LH cows showed significantly higher propionate levels and lower acetate-to-propionate ratios (*p* < 0.05; Table 2). No significant differences were observed in oxidative stress, inflammatory, and immunological biomarkers between the two groups (*p* > 0.05; Table 3). However, serum TC, HDL-C, and LDL-C levels were significantly higher in LH cows, whereas INS and GCG levels were significantly lower compared to LL cows (*p* < 0.05; Table 3). These results indicate that the two groups of cows were successfully selected and have good physiological indicators.

**Table 1:** Physiological parameters of LH and LL cows.

Performance	Group		SEM	*p*-value	95%CI	Effect sizes^6^
	LH	LL				
MF, %	3.72	3.76	0.15	0.75	−0.31, 0.44	0.24
MP, %	3.35	3.37	0.11	0.83	−0.17, 0.30	0.32
ML, %	4.73	4.76	0.17	0.82	−0.27, 0.45	0.32
MSNF, %	8.85	8.92	0.29	0.81	−0.52, 0.74	0.22
MTS, %	12.69	12.56	0.26	0.70	−0.45,1.55	0.69
MFP, °C	−0.53	−0.56	0.02	0.23	−0.05, 0.05	−0.03
MD, g/cm^3^	1.03	1.03	0.005	0.91	−0.002,0.004	0.53
EC, mS/cm	5.02	4.88	0.12	0.25	−0.60, 0.44	−0.19
Ash, %	0.78	0.79	0.01	0.86	−0.05, 0.06	0.09
MY, kg/d	55.84	37.41	1.74	<0.001	14.45, 23.81	5.16
MFY^1^, kg/d	2.08	1.38	0.10	<0.001	0.48, 1.00	3.58
MPY^2^, kg/d	1.87	1.26	0.04	<0.001	0.46, 0.87	4.15
ECM^3^, kg/d	59.44	39.21	2.23	<0.001	14.78, 26.38	4.48
FCM^4^, kg/d	57.75	39.78	1.13	<0.001	11.01, 19.55	4.52
DMI, kg/d	25.40	24.03	0.46	0.13	−0.52, 3.85	0.96
FE^5^, kg/kg	1.73	1.24	0.14	<0.001	0.39, 0.66	4.87
305-day MY, kg/d	49.29	35.76	1.47	<0.001	15.42, 22.54	6.73
Historical average 305-day MY, kg/d	47.53	38.66	2.36	<0.01	5.85, 15.72	2.76
BCS	3.34	3.19	0.24	0.53	−0.79, 0.42	−0.39
Parity	5.75	6.25	0.40	0.23	−0.68, −0.06	−0.37
Age, y	8.63	8.75	0.25	0.91	−0.65, 0.58	−0.07
DIM, d	127.25	134.50	3.54	0.14	−0.04, 0.03	−0.01
RpH	6.03	6.40	0.12	<0.01	−0.56, −0.03	−1.38

LH, long-lived high-yielding cows; LL, long-lived low-yielding cows; SEM, standard error of the mean; CI, confidence interval; MF, milk fat; MP, milk protein; ML, milk lactose; MSNF, milk solid-not-fat; MTS, milk total solids; MFP, milk freezing point; MD, milk density; EC, electrical conductivity; Ash, ash content; MY, milk yield; MFY, milk fat yield; MPY, milk protein yield; ECM, energy-corrected milk; FCM, fat-corrected milk; DMI, dry matter intake. DMI was defined as the total dry matter intake from all feed components; FE, feed efficiency; FE was defined as MY/ DMI; BCS, body condition score; DIM, days in milk; RpH, rumen pH.

1 MFY (kg/d) = MY (kg/d) × MF (%).

2 MPY (kg/d) = MY (kg/d) × MP (%).

3 FCM (kg/d) = [0.432 + 0.162 × milk fat (%)] × milk yield (kg/d).

4 ECM (kg/d) = 0.327 × milk yield (kg/d) + 12.95 × fat yield (kg/d) + 7.65 × protein yield (kg/d) (Tyrrell and Reid, 1965).

5 FE (kg/kg) was defined as MY (kg/d)/DMI (kg/d).

6 Between-group effect sizes are expressed as Cohen’s d (standardized mean difference) based on the adjusted groups contrast. Effects of covariates (parity, age, and DIM) are reported as regression coefficients (β) with 95% CI from the multivariable models.

**Table 2:** Comparison of rumen VFAs between LH and LL cows.

Performance	Group		SEM	*P*-value	95%CI	Effect sizes^1^
	LH	LL				
Total VFA, mmol/L	154.74	154.24	3.15	0.94	−15.46, 16.17	0.028
Acetate, mmol/L	70.20	73.03	1.48	0.39	−3.56, −11.65	−0.56
Propionate, mmol/L	41.61	34.81	1.34	<0.05	1.19, 15.5	1.48
Acetate/propionate	1.83	2.11	0.09	<0.05	−0.82, −0.14	1.80
Isobutyrate, mmol/L	6.26	6.54	0.38	0.32	−2.02, 0.75	−0.58
Butyrate, mmol/L	20.03	20.09	0.56	0.25	−5.84, 1.19	−0.84
Isovalerate, mmol/L	6.56	6.33	0.33	0.73	−2.03, 1.30	−0.28
Valerate, mmol/L	8.16	8.23	0.48	0.91	−1.96, 2.36	0.12
4-methylvalerate, mmol/L	0.03	0.03	0.004	0.79	−0.02, 0.02	0.14
Caproate, mmol/L	1.85	3.13	0.27	0.08	−2.75, 0.03	−1.23

LH, long-lived high-yielding cows; LL, long-lived low-yielding cows; Total VFA, total volatile fatty acid; SEM, standard error of the mean; CI, confidence interval.

1 Between-group effect sizes are expressed as Cohen’s d (standardized mean difference) based on the adjusted groups contrast.

**Table 3:** Comparison of serum biochemical parameters between LH and LL cows.

Performance	Group		SEM	*P*-value	95%CI	Effect sizes^1^
	LH	LL				
Serum biochemical parameters						
TP, g/L	71.06	74.49	1.88	0.22	−10.18, 1.99	−0.85
ALB, g/L	43.95	44.36	1.34	0.84	−5.04, 5.12	0.01
GLB, g/L	27.11	30.13	1.38	0.14	−8.81, 0.54	−1.11
ALB/GLB	1.65	1.50	0.10	0.31	−0.14, 0.58	0.76
AST, U/L	85.78	86.06	3.05	0.95	−4.01, 13.81	0.69
ALT, U/L	43.02	39.21	1.40	0.08	−0.48, 10.00	1.14
ALP, U/L	233.19	216.97	12.22	0.36	−35.31, 52.82	0.25
TBA, μmol/L	4.88	5.77	0.39	0.12	−2.31, 0.64	−0.71
γ-GGT, U/L	32.00	32.62	2.77	0.87	−11.78, 9.56	−0.13
BUN, mmol/L	5.54	5.14	0.30	0.37	−0.64, 1.67	0.56
CRE, μmol/L	75.61	79.56	3.09	0.38	−9.81, 10.90	0.07
LDH, U/L	678.83	721.22	25.83	0.27	−132.97, 63.40	−0.45
GLU, mmol/L	4.17	4.57	0.23	0.23	−1.07, 0.60	−0.36
TC, mmol/L	4.78	4.00	0.20	<0.05	0.26, 1.43	1.25
TG, mmol/L	0.48	0.50	0.01	0.25	−0.07, 0.03	−0.57
HDL-C, mmol/L	2.22	1.96	0.07	<0.05	0.00, 0.51	1.27
LDL-C, mmol/L	2.47	1.95	0.13	<0.05	0.16, 0.93	1.11
NEFA, μmol /L	396.50	426.02	31.50	0.52	−15.93, 8.02	−0.42
BHB, μmol /L	736.10	760.24	34.27	0.63	−180.73, 72.65	−0.54
Oxidative stress biomarkers						
SOD, U/mL	105.75	106.64	3.33	0.85	−16.29, 7.75	−0.45
GSX-PX, U/mL	59.67	60.13	1.30	0.80	−11.75, 6.41	−0.37
T-AOC, mmol/L	1.88	1.90	0.07	0.77	−1.70, 0.83	−0.44
T-GSH, μmol/L	3.66	3.71	0.08	0.60	−1.11, 1.34	0.12
CAT, U/mL	5.96	6.22	0.12	0.67	−1.18, 0.65	−0.37
MDA, μmol/L	3.66	3.71	0.05	0.47	−0.20, 0.02	−0.62
ProtC, μmol /L	1.63	1.65	0.07	0.74	−0.19, 0.18	−0.03
Inflammatory and immunological markers						
IL-1β, pg./mL	8.30	8.39	0.71	0.93	−2.38, 2.86	0.11
IL-6, pg./mL	21.53	22.32	1.51	0.71	−6.14, 5.35	−0.09
IL-10, pg./mL	11.89	11.93	0.64	0.96	−2.92, 1.81	−0.30
HPT, μg/mL	39.80	36.57	2.33	0.34	−1.98, 3.57	0.36
TNF-α, pg./mL	16.78	15.40	1.10	0.39	−2.48, 5.91	0.51
SAA, μg/mL	25.78	24.65	1.31	0.76	−7.32, 9.02	0.13
IgG, g/L	21.86	22.21	0.79	0.76	−1.99, 0.77	−0.56
IgA, g/L	0.81	0.82	0.03	0.76	−0.06, 0.05	−0.51
Endocrine markers						
GH, ng/mL	1.98	2.40	0.12	0.05	−0.88, 0.01	−1.17
IGF-1, ng/mL	41.24	48.62	2.23	0.08	−16.09, 1.21	−1.09
PRL, μIU/L	1303.89	1333.29	54.17	0.71	−238.04, 178.11	−0.18
COR, pg./mL	39.12	41.37	1.62	0.34	−9.70, 2.14	−0.81
T3, ng/mL	1.97	1.89	0.11	0.63	−0.32, 0.51	0.28
T4, ng/mL	50.50	61.78	5.71	0.18	−30.10, 6.58	−0.81
INS, mIU/L	11.47	15.71	1.19	<0.05	−8.95, −1.09	−1.62
GCG, pg./mL	60.67	77.46	4.44	<0.05	−34.43, −4.78	−1.67

LH, long-lived high-yielding cows; LL, long-lived low-yielding cows; SEM, standard error of the mean; CI, confidence interval; TP, total protein; ALB, albumin; GLB, globulin; AST, aspartate aminotransferase; ALT, alanine aminotransferase; ALP, alkaline phosphatase; TBA, total bile acids; γ-GGT, gamma-glutamyl transferase; BUN, blood urea nitrogen; CRE, creatinine; LDH, lactate dehydrogenase; GLU, glucose yield; TC, total cholesterol; TG, triglycerides; HDL-C, high-density lipoprotein cholesterol; LDL-C, low-density lipoprotein cholesterol; NEFA, non-esterified fatty acids; BHB, beta-hydroxybutyrate; SOD, superoxide dismutase; GSH-PX, glutathione peroxidase; T-AOC, total antioxidant capacity; T-GSH, total glutathione; CAT, catalase; MDA, malondialdehyde; PC, protein carbonyl; IL-1β, interleukin-1 beta; IL-6, interleukin-6; IL-10, interleukin-10; HPT, haptoglobin; TNF-α, tumor necrosis factor-alpha; SAA, serum amyloid A; IgG, immunoglobulin G; IgA, immunoglobulin A; GH, growth hormone; IGF-1, insulin-like growth factor 1; PRL, prolactin; COR, cortisol; T3, triiodothyronine; T4, thyroxine; INS, insulin; GCG, glucagon.

1 Between-group effect sizes are expressed as Cohen’s d (standardized mean difference) based on the adjusted groups contrast.

### 3.2 Structural differences between the milk microbiomes of LH and LL dairy cows

**Figure 1:**
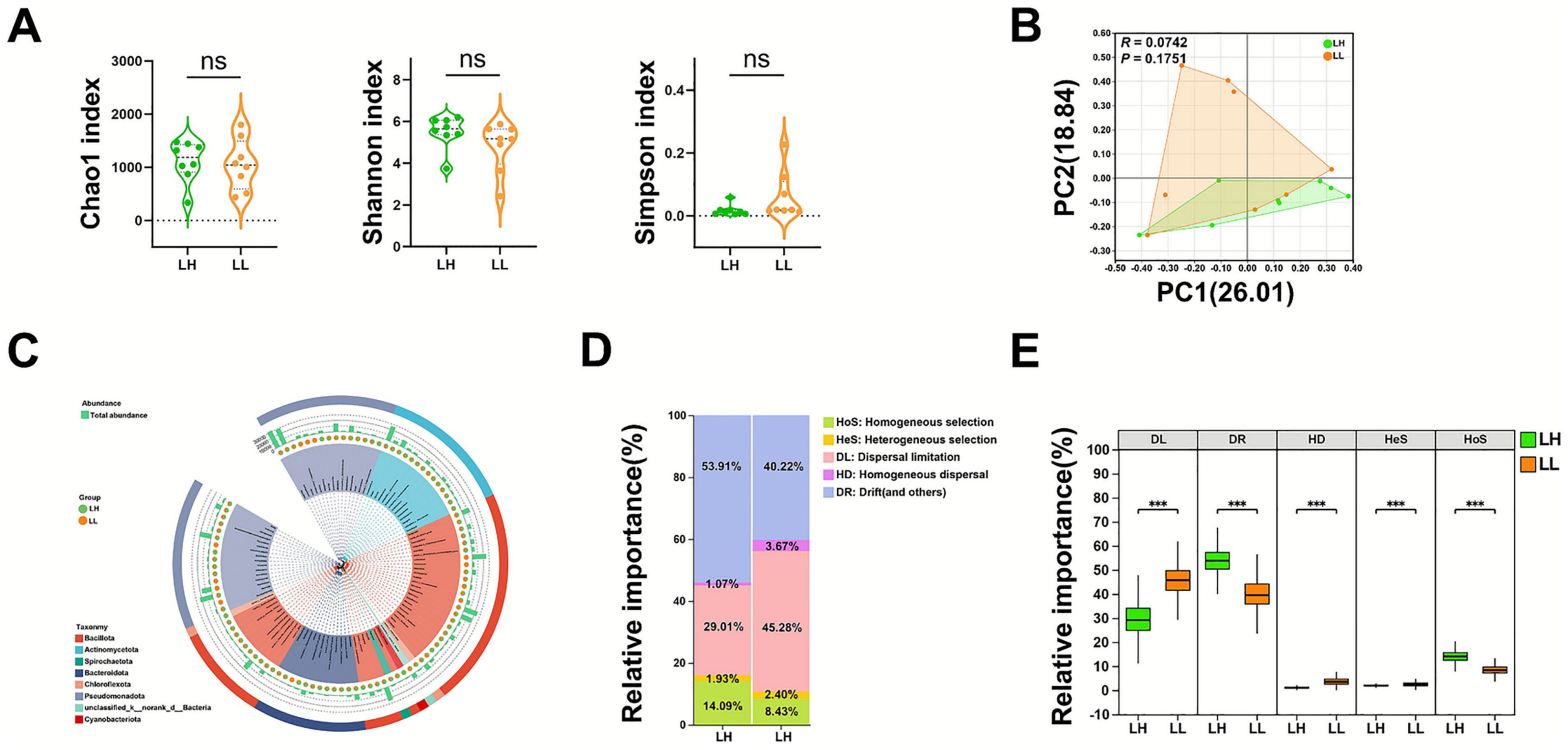
Comparison of milk microbiota diversity and community structure between LH and LL cows. **(A)** Comparison of α-diversity indices (Chao1, Shannon, and Simpson) of the milk microbiota between LH and LL groups at the genus level (*n* = 8 per group). Error bars represent mean ± SEM. *p* values were calculated using the Wilcoxon rank-sum test. **(B)** PCoA of milk microbiota at the genus level between LH and LL groups based on Bray–Curtis dissimilarity (*n* = 8 per group). Dissimilarity was analyzed using ANOSIM with 999 permutations. **(C)** Phylogenetic tree of the milk microbiota at the genus level, constructed using the maximum-likelihood method. **(D)** Proportional estimation and **(E)** differences in the ecological processes governing milk microbiota assembly between the two groups (*n* = 8 per group), inferred using the null-model framework implemented in iCAMP. Error bars represent mean ± SEM. *p* values were calculated using the Wilcoxon rank-sum test; ***p < 0.001. SEM, Standard error of the mean; PCoA, Principal coordinate analysis; ANOSIM, Analysis of similarities; iCAMP, Individual-level community assembly mechanisms based on phylogenetic bin-null model analyses.

The milk microbiota diversity of LH cows was compared with that of LL cows, and no significant differences were observed in the ACE (*p* = 0.8064), Chao1 (*p* = 0.7868), Shannon (*p* = 0.1852), and Simpson (*p* = 0.2717) indices (Figure 1A). For *β*-diversity (Figure 1B), PCoA revealed no distinct separation of the milk microbiota between the two groups (*R* = 0.0742, *p* = 0.1751). Phylogenetic trees of the milk microbiota communities in LH and LL cows were constructed (Figure 1C). Using iCAMP (infer Community Assembly Mechanisms by Phylogenetic-bin-based null model analysis; a phylogenetic-binning, null-model framework that quantifies the relative contributions of community assembly processes), we detected marked differences between groups. The milk microbiota of LH cows showed significantly higher contributions from homogeneous selection (deterministic, non-random filtering under similar environmental conditions leading to community convergence; 14.09% vs. 8.43% in LL cows; *p* < 0.001) and from ecological drift plus other undominated processes (stochastic, directionless fluctuations driven by random birth–death events and chance colonization when selection/dispersal effects are weak; 53.91% vs. 40.22%; *p* < 0.001). In contrast, LH cows exhibited significantly lower contributions from dispersal limitation (restricted dispersal or spatial barriers limiting taxa arrival, thereby enhancing non-random turnover and between-community divergence; 29.01% vs. 45.28%; p < 0.001), homogenizing dispersal (high dispersal rates that reduce turnover and promote community similarity; 1.07% vs. 1.93%; *p* < 0.001), and heterogeneous selection (deterministic filtering imposed by environmental heterogeneity that drives non-random community differentiation; 1.93% vs. 3.67%; *p* < 0.001) compared with LL cows (Figures 1D,E). These findings suggest that the milk microbiota in LH cows exhibited greater homogeneity, reduced dispersal limitations, and decreased heterogeneous selection. Overall, the microbial community of LH cows exhibited lower internal variability, and greater homogeneity and stability compared with that of LL cows.

### 3.3 Differences in taxonomy and function of milk microbiota

**Figure 2:**
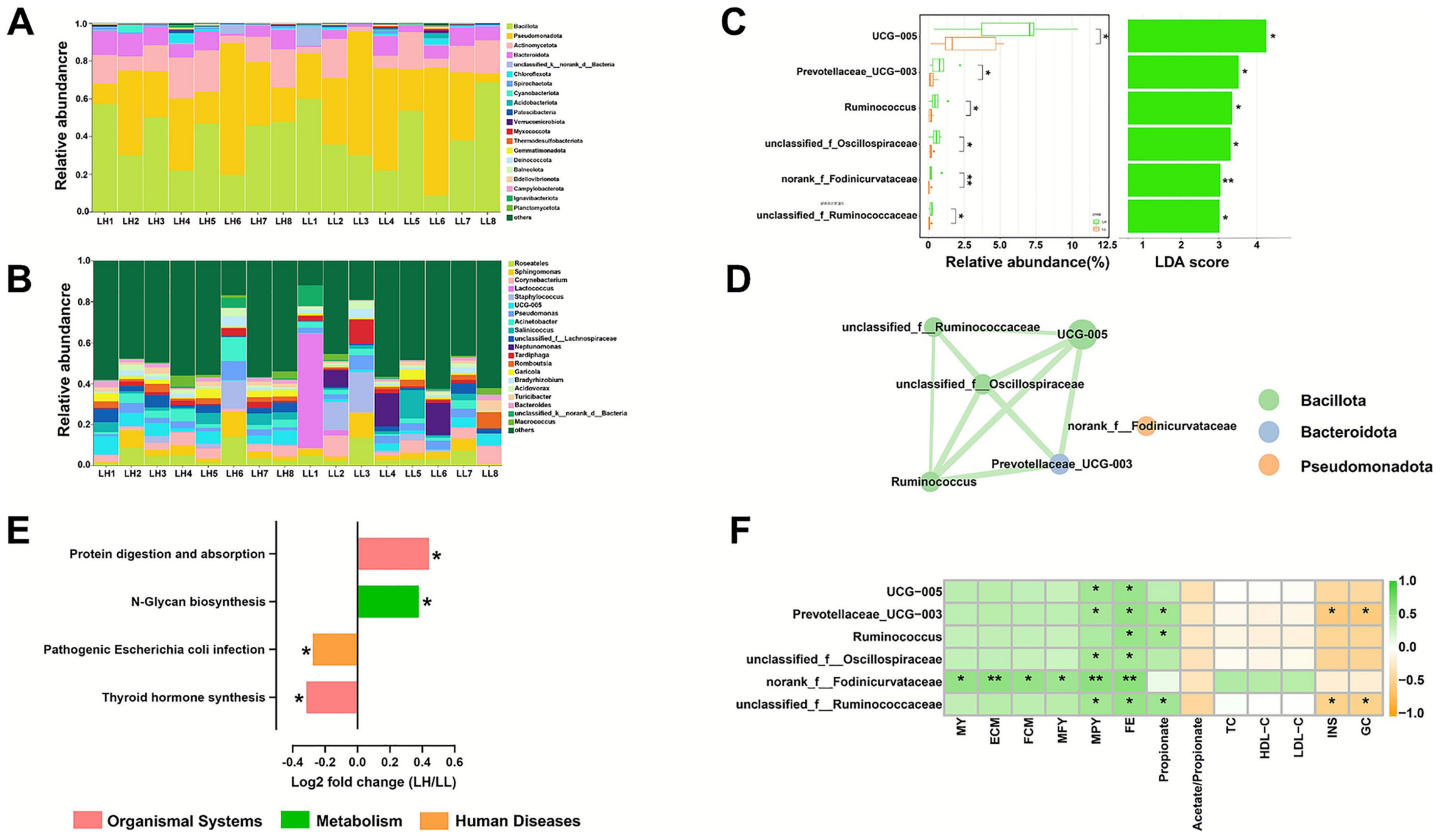
Comparison of milk microbiota composition between LH and LL cows. Relative abundances of the milk microbiota at the phylum **(A)** and genus **(B)** levels. **(C)** Comparison of the relative abundances of differentially enriched genera (LDA > 2, *p* < 0.05) between the two groups (*n* = 8 per group). Error bars represent mean ± SEM. *p* values were calculated using the Wilcoxon rank-sum test; *p < 0.05; **p < 0.01. **(D)** Co-occurrence network of differentially enriched genera. Edges denote Spearman’s correlation coefficients; green lines indicate positive correlations (|*r*| > 0.50 and *p* < 0.05). **(E)** Differentially enriched KEGG evel 3 pathways in the milk microbiota predicted using PICRUSt2 (LDA > 2, *p* < 0.05) between the two groups (*n* = 8 per group). *p* values were calculated using the Wilcoxon rank-sum test; *p < 0.05. **(F)** Spearman’s correlations between differentially enriched genera and phenotypic traits. *p < 0.05; **p < 0.01. SEM, standard error of the mean; PICRUSt2, phylogenetic investigation of communities by reconstruction of unobserved states; KEGG, Kyoto Encyclopedia of Genes and Genomes. MY, milk yield; MFY, milk fat yield; MPY, milk protein yield; ECM, energy-corrected milk; FCM, fat-corrected milk; FE, feed efficiency; TC, total cholesterol; HDL-C, high-density lipoprotein cholesterol; LDL-C, low-density lipoprotein cholesterol; INS, insulin; GCG, glucagon.

The dominant bacterial phyla (relative abundance >1%; Figure 2A) in the milk microbiota were Bacillota (39.73 ± 4.19%), Pseudomonadota (35.42 ± 5.09%), Actinomycetota (12.90 ± 1.79%), Bacteroidota (7.06 ± 0.99%), and unclassified k norank d Bacteria (1.23 ± 0.67%). The predominant bacterial genera (relative abundance >2%; Figure 2B) included *Roseateles* (4.82 ± 1.03%), *Sphingomonas* (4.39 ± 0.91%), *Corynebacterium* (3.94 ± 0.75%), *Lactococcus* (3.70 ± 3.48%), *Staphylococcus* (3.68 ± 1.54%), *UCG-005* (3.64 ± 0.69%), *Pseudomonas* (3.46 ± 0.56%), *Acinetobacter* (3.20 ± 0.67%), *Salinicoccus* (2.88 ± 0.86%), *unclassified f Lachnospiraceae* (2.59 ± 0.55%), *Tardiphaga* (2.15 ± 0.68%), and *Romboutsia* (2.07 ± 0.48%). Comparison of bacterial genera with relative abundances greater than 0.1% identified six differential genera, all enriched in the LH group (Figure 2C). These genera included *UCG-005* (LDA = 4.1881, *p* = 0.0460), *Prevotellaceae UCG-003* (LDA = 3.4331, *p* = 0.0357), *Ruminococcus* (LDA = 3.2455, *p* = 0.0357), *unclassified f Oscillospiraceae* (LDA = 3.2257, *p* = 0.0460), *norank f Fodinicurvataceae* (LDA = 3.0085, *p* = 0.0063), and *unclassified f Ruminococcaceae* (LDA = 3.0079, *p* = 0.0456).

A co-occurrence network was constructed for the differential genera based on Spearman correlation coefficients (|*r*| > 0.50, *p* < 0.05). The analysis revealed that *norank f Fodinicurvataceae* did not exhibit any connections with the other five genera (|*r*| < 0.50, *p* > 0.05), whereas positive associations were observed among the remaining five genera (Figure 2D; |*r*| > 0.50, *p* < 0.05). Functional prediction using PICRUSt2 revealed significant differences in KEGG level 3 pathways between the two groups. Protein digestion and absorption (*p* = 0.0313) and N-glycan biosynthesis (*p* = 0.0240) were significantly enriched in the LH group, whereas thyroid hormone synthesis (*p* = 0.0181) and pathogenic *E. coli* infection (*p* = 0.0406) were significantly enriched in the LL group (Figure 2E). Correlation analysis between these differential bacterial taxa and phenotypic traits (Figure 2F) revealed that *norank f Fodinicurvataceae* was significantly correlated with all six production traits (MY, ECM, FCM, MFY, MPY, and FE; |*r*| > 0.50, *p* < 0.05). In addition, *UCG-005*, *Prevotellaceae UCG-003*, *unclassified f Oscillospiraceae*, *norank f Fodinicurvataceae*, and *unclassified f Ruminococcaceae* were positively correlated with MPY and FE (|*r*| > 0.50, *p* < 0.05). *Prevotellaceae UCG-003*, *Ruminococcus*, and *norank f Fodinicurvataceae* were positively correlated with rumen propionate levels (|*r*| > 0.50, *p* < 0.05), whereas *Prevotellaceae UCG-003* and *unclassified f Ruminococcaceae* were negatively correlated with serum INS and GCG levels (|*r*| > 0.50, *p* < 0.05).

### 3.4 Metabolomic differences in the milk of LH versus LL dairy cows

**Figure 3:**
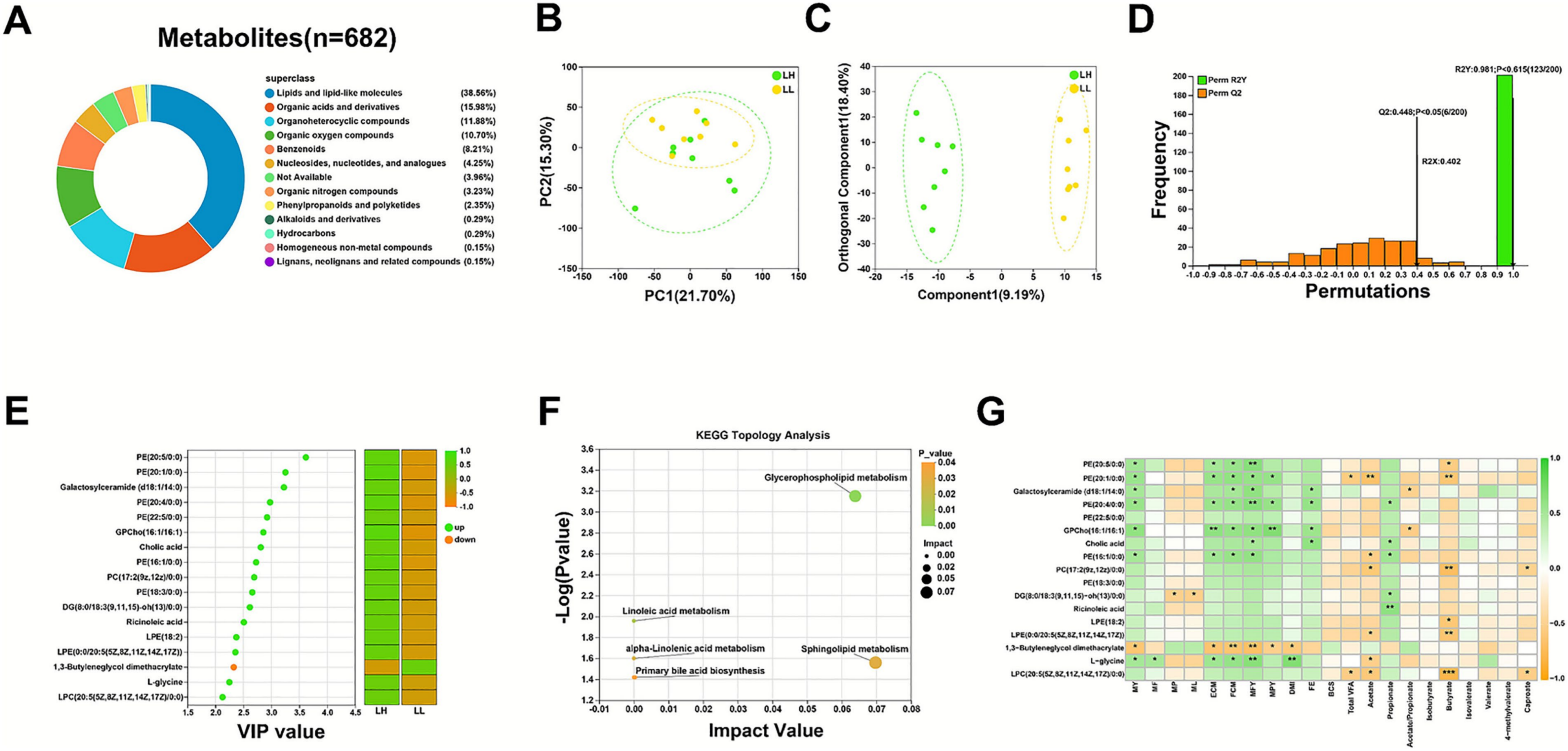
Comparison of metabolomic profiles between LH and LL cows. **(A)** Pie chart showing the percentages of identified metabolites in each chemical class. **(B)** PCA showing the distribution of milk metabolic profiles between LH and LL cows (*n* = 8 per group). **(C)** OPLS-DA of milk metabolites from LH and LL groups. **(D)** OPLS-DA permutation test assessing the model’s goodness of fit, with *R*^2^*Y* = variance explained in the response variable (*Y*); *Q*^2^ = predictive ability of the model (*n* = 8 per group). **(E)** Identification of differential metabolites (*n* = 8 per group) in milk (VIP > 1, LH/LL fold change >1.05). **(F)** Bubble plot showing significantly enriched metabolic pathways based on topological pathway analysis (FDR-adjusted *p* < 0.05). **(G)** Spearman’s correlation heatmap between differential milk metabolites and phenotypic traits. *p < 0.05; **p < 0.01; ***p < 0.001. LH, long-lived high-yielding cows; LL, long-lived low-yielding cows; PCA, principal component analysis; OPLS-DA, orthogonal partial least squares discriminant analysis; VIP, variable importance in projection; PE, phosphatidylethanolamine; PC, phosphatidylcholine; DG, diglyceride; LPE, lysophosphatidylethanolamine; GPCho, glycophosphocholine; LPC, lysophosphatidylcholine; MY, milk yield; MFY, milk fat yield; MPY, milk protein yield; ECM, energy-corrected milk; FCM, fat-corrected milk; FE, feed efficiency; TC, total cholesterol; HDL-C, high-density lipoprotein cholesterol; LDL-C, low-density lipoprotein cholesterol; INS, insulin; GCG, glucagon; FDR, false discovery rate.

The QC samples exhibited good stability and high consistency, indicating that the data quality meets the requirements for metabolomics analysis (Supplementary Tables S1A, S1B). A total of 682 milk metabolites were characterized across the two dairy cow groups (Figure 3A). Among them, lipids and lipid-like molecules were predominant (263, 38.56%), followed by organic acids and derivatives (109, 15.98%), organoheterocyclic compounds (81, 11.88%), organic oxygen compounds (73, 10.70%), and benzenoids (56, 8.21%). Other metabolite classes included nucleosides, nucleotides and analogs (29, 4.25%), unclassified metabolites (27, 3.96%), organic nitrogen compounds (22, 3.23%), as well as phenylpropanoids and polyketides (16, 2.35%). To assess metabolic distinctions between the two groups, PCA and OPLS-DA were conducted (Figures 3B,C). The resulting model demonstrated strong explanatory power (R2Y = 0.981) and moderate predictive reliability (Q2 = 0.448; Figure 3D). Seventeen metabolites differed significantly between the groups, with 16 enriched in the LH group (VIP > 1, LH/LL fold change >1.05; Figure 3E). Pathway enrichment analysis revealed significant associations with glycerophospholipid metabolism (FDR-adjusted *p* = 0.0021), glycerophospholipid metabolism (FDR-adjusted *p* = 0.0021), linoleic acid metabolism (FDR-adjusted *p* = 0.0220), sphingolipid metabolism (FDR-adjusted *p* = 0.0331), primary bile acid biosynthesis (FDR-adjusted *p* = 0.0352), and alpha-linolenic acid metabolism (FDR-adjusted *p* = 0.0377; Figure 3F).

Correlation analysis indicated that seven metabolites, including PE (20:5/0:0), PE (20:1/0:0), galactosylceramide (d18:1/14:0), PE (20:4/0:0), GPCho (16:1/16:1), PE (16:1/0:0), and l-glycine, were positively associated with MY (|*r*| > 0.50, *p* < 0.05). Furthermore, PE (20, 4/0:0), cholic acid, PE (16, 1/0, 0), DG (8:0/18:3 (9, 11, 15)-oh(13)/0:0), and ricinoleic acid showed positive correlations with rumen propionate levels (|*r*| > 0.50, *p* < 0.05; Figure 3G). In contrast, PE (20, 1/0, 0), GPCho (16, 1/16, 1), PC (17:2 (9z, 12z)/0:0), LPE (0:0/20:5 (5Z, 8Z, 11Z, 14Z, 17Z)), and LPC (20:5 (5Z, 8Z, 11Z, 14Z, 17Z)/0:0) were negatively correlated with serum INS and GCG (|*r*| > 0.50, *p* < 0.05; Figure 3G).

### 3.5 Correlation analysis between multiple omics and phenotypes

**Figure 4:**
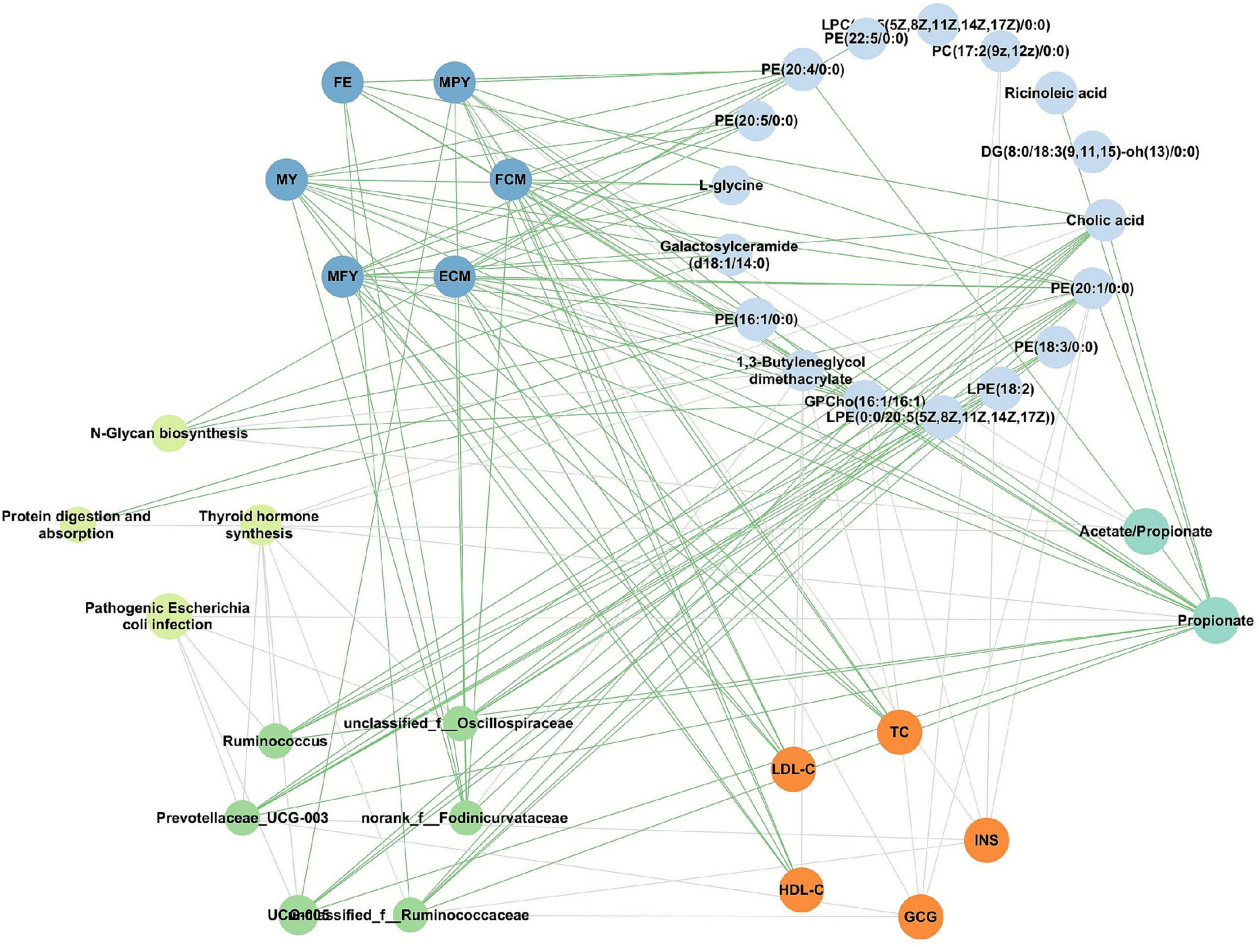
Associations among multi-omics data and phenotypic traits. Spearman correlation network illustrating the relationships among differential phenotypic traits, rumen fermentation parameters, serum lipids, milk microbial genera, microbial functions, and milk metabolites. Edges between nodes represent Spearman correlations. Green lines indicate positive and gray lines indicate negative correlations (|*r*| > 0.50, *p* < 0.05). PE, phosphatidylethanolamine; PC, phosphatidylcholine; DG, diglyceride; LPE, lysophosphatidylethanolamine; GPCho, glycophosphocholine; LPE, lysophosphatidylethanolamine; LPC, lysophosphatidylcholine; MY, milk yield; MFY, milk fat yield; MPY, milk protein yield; ECM, energy-corrected milk; FCM, fat-corrected milk; FE, feed efficiency; TG, triglyceride; TC, total cholesterol; HDL-C, high-density lipoprotein cholesterol; LDL-C, low-density lipoprotein cholesterol; INS, insulin; GCG, glucagon.

We further analyzed the associations among differential phenotypes, rumen fermentation parameters, serum biomarkers, bacterial genera in milk, microbialfunctions, and milk metabolites (Figure 4). Milk PE (18:3/0:0) was positively correlated with milk *Prevotellaceae UCG-003* and *UCG-005* (|*r*| > 0.50, *p* < 0.05), while milk LPE (0:0/20:5 (5Z, 8Z, 11Z, 14Z, 17Z)) was positively correlated with milk *Prevotellaceae UCG-003* (*r* = 0.5504, *p* = 0.0272). Moreover, *unclassified f Oscillospiraceae*, *Prevotellaceae UCG-003*, *Ruminococcus* and *UCG-005* in milk were negatively correlated with the predicted microbial function pathogenic *E. coli* infection (|*r*| > 0.50, *p* < 0.05). However, no significant correlations were found between microbial functions and milk production traits.

## 4 Discussion

The milk microbiome and metabolome are closely associated with mammary gland health and directly influence the persistent productivity of dairy cows (Wang et al., 2020). Therefore, in this study, we investigated the milk microbiome and metabolome of LH and LL cows to examine their associations with phenotypic traits and to clarify their potential roles in supporting consistent milk production.

In this study, the milk microbiome of LH cows showed lower homogeneous dispersal and heterogeneous selection, but higher homogeneous selection and ecological drift. Frequent milk turnover may promote a more homogeneous and stable milk microbiome, in which microorganisms within the community are more likely to interact and disperse through random processes (Zhu et al., 2021). This phenomenon may reflect a unique physiological and metabolic adaptation in LH cows, helping to maintain a balanced milk microbiome that supports mammary health and stability of the microbial community. This study found that, among the enriched taxa, *Prevotellaceae UCG-003* was more abundant in LH cows. Previous studies have shown that *Prevotellaceae UCG-003* was present at lower levels in the feces of cows with clinical mastitis (Wang et al., 2022a), but was enriched in the milk of subclinical mastitis cows supplemented with inulin, where its abundance was positively correlated with MY (Wang et al., 2022b). Therefore, the enrichment of *Prevotellaceae UCG-003* in LH milk may be associated with a healthier gastrointestinal microbiome and improved mammary health. *Ruminococcus* and *unclassified f Ruminococcaceae* were also enriched in the LH milk. *Ruminococcus* may have a greater ability to migrate from the GI tract to the mammary gland (Young et al., 2015). The enrichment of these two genera in milk suggests that their transfer from the GI tract to the mammary gland is more frequent in LH cows, although this hypothesis requires further investigation. *UCG-005* within the Oscillospiraceae was the most abundant genus enriched in the milk of LH cows. This genus had previously been identified as dominant in early lactation milk (Zhu et al., 2023). *UCG-005* in the feces of calves has been shown to inhibit the pathogenic bacteria *Mannheimia* and *Pasteurella* (Uddin et al., 2024). The enrichment of *UCG-005* and *unclassified f Oscillospiraceae* in the milk of LH cows may be associated with reduced mastitis pathogens and improved mammary health. Furthermore, *Prevotellaceae UCG-003*, *unclassified f Oscillospiraceae*, *Ruminococcus*, and *UCG-005* were negatively associated with microbial functions related to pathogenic *E. coli* infection, suggesting that these bacteria may play a protective role in inhibiting pathogenic *E. coli* infection in the mammary gland. However, given the potential false-positive risk of Spearman correlations in small sample sizes, future studies with larger cohorts are needed to further validate these findings.

Previous studies have shown that a high MY does not necessarily equate to milk with a higher nutritional value. Milk protein concentration has been reported to be negatively correlated with MY (Morton et al., 2016). To further explore potential nutritional differences, we conducted untargeted metabolomics and found that LH cows exhibited enrichment in PE (20:5/0:0), LPE (0:0/20:5 (5Z, 8Z, 11Z, 14Z, 17Z)), LPC (20:5 (5Z, 8Z, 11Z, 14Z, 17Z)/0:0), PE (22:5/0:0), DG (8:0/18:3(9, 11, 15)-oh(13)/0:0), and PE (18:3/0:0). In aging dairy cows, omega-3 polyunsaturated fatty acids (PUFAs), such as Docosapentaenoic acid (DPA), are typically downregulated (Sheedy et al., 2025). The LH cows may maintain a healthier physiological state compared to LL cows, preserving higher levels of blood omega-3 PUFAs, which are utilized by the mammary gland as precursors for milk omega-3 PUFAs production. Eicosapentaenoic acid (EPA) has been shown to reverse INS resistance (Sun et al., 2021) and to prevent cardiovascular disease (Sweeney et al., 2023). DPA exerts lipid-lowering and glucose-regulating effects (Hirako et al., 2023), whereas *α*-linolenic acid (ALA) demonstrates antihypertensive, anti-atherosclerotic, and cardioprotective properties (Bertoni et al., 2023). Moreover, omega-3 PUFAs constitute approximately 35% of the total fatty acids in brain (Dighriri et al., 2022), and higher maternal omega-3 PUFA levels may be associated with enhanced neurodevelopment in offspring (Zou et al., 2021). These omega-3 PUFAs can also positively influence mood and behavior, and reduce adverse psychological symptoms, such as stress, anxiety, and depression (Kiecolt-Glaser et al., 2011; Larrieu and Layé, 2018; Zou et al., 2021). A certain proportion of omega-3 PUFAs in milk exists in the PLs fraction of milk fat globules (Castañeda-Gutiérrez et al., 2007; Ting et al., 2015) and omega-3 PUFAs in human milk Phospholipids (PLs) show high bioavailability (Gumus and Gharibzahedi, 2021). Furthermore, omega-3 PUFAs in PLs exhibit superior anti-obesity and lipid-lowering effects, as well as improvements in oxidative stress in the central nervous system (Che et al., 2018; Li et al., 2018). Therefore, omega-3 PUFAs enriched in the PLs fraction of milk from LH cows may exhibit greater nutritional potential compared with those from LL cows. However, because supervised learning methods such as OPLS-DA inherently carry a theoretical risk of overfitting, which may exaggerate group separation, and because of the limited sample size in this study, further validation using quantitative omics approaches is warranted to strengthen the conclusions.

In addition, PE (18:3/0:0) in milk was positively correlated with milk *Prevotellaceae UCG-003*, *unclassified f Oscillospiraceae*, and *UCG-005*. Milk LPE (0:0/20:5 (5Z, 8Z, 11Z, 14Z, 17Z)) was also positively correlated with milk *Prevotellaceae UCG-003*. Notably, the abundance of *Prevotellaceae UCG-003* in the rumen has been reported to be associated with ALA (Conte et al., 2022), and the abundance of Oscillospiraceae in mouse feces was found to increase with dietary lipid supplementation (Koontanatechanon et al., 2022). ALA is known to inhibit the growth of mastitis-causing pathogens, including *E. coli*, *Staphylococcus epidermidis*, and *S. aureus*, by suppressing bacterial fatty acid (FA) synthesis (Yadav et al., 2019), while EPA may inhibit *Bacillus cereus* and *S. aureus* by disrupting their cell membranes (Kaithwas et al., 2011; Yadav et al., 2019). Therefore, the elevated levels of specific omega-3 PUFAs in the milk of LH cows may be associated with a reduction in pathogenic microorganisms and an enrichment of beneficial microbes in the mammary gland, thereby supporting the maintenance of a healthier mammary microbial ecosystem.

Although potential confounding factors such as feed, management, and lactation stage were carefully controlled in this study, the sample size may still limit the generalizability of the findings. In addition, this study did not systematically assess the sources of variability in the milk microbiome, nor did it include negative controls or apply dedicated contaminant identification and removal approaches. These limitations may affect the precise interpretation of milk microbiome composition and between-group differences (Moossavi et al., 2020; Moossavi et al., 2021; Dean et al., 2024). Therefore, the conclusions drawn from this study should be interpreted with caution. Future studies should increase sample size and incorporate negative controls, batch-effect management, and more rigorous decontamination strategies to improve the reliability and interpretability of milk microbiome data in cows with different levels of production longevity.

## 5 Conclusion

The results of this multi-omics study reveal significant differences in the milk microbiome and metabolome between LH and LL dairy cows. Specifically, the milk of LH cows was enriched in *Prevotellaceae UCG-003*, *Ruminococcus*, *unclassified f Ruminococcaceae*, *unclassified f Oscillospiraceae*, *norank f Fodinicurvataceae*, and *UCG-005*, as well as in omega-3 PUFA-PLs, such as PE (20:5/0:0), PE (22:5/0:0), PE (18:3/0:0), DG (8:0/18:3 (9, 11, 15)-oh (13)/0:0), LPE (0:0/20:5 (5Z, 8Z, 11Z, 14Z, 17Z)), and LPC (20:5 (5Z, 8Z, 11Z, 14Z, 17Z)/0:0). These findings provide new insights into the integrated milk microbial and metabolic traits of long-lived cows possessing sustained high production capacity. Understanding the unique physiological states of LH cows may help set strategies to improve the health and productivity of aging dairy cows, while also enhancing the nutritional value of their milk.

## Data Availability

The datasets presented in this study can be found in online repositories. The names of the repository/repositories and accession number(s) can be found at: https://www.ncbi.nlm.nih.gov/, PRJNA1307952.

## Footnotes

1
http://www.hmdb.ca/

2
https://www.genome.jp/kegg/

3
https://metlin.scripps.edu/

4
https://www.kegg.jp/kegg/pathway.html

## Glossary

ALA: alpha-linolenic acid
ALB: albumin
ALP: alkaline phosphatase
ALT: alanine aminotransferase
Ash: ash content
ASVs: amplicon sequence variants
AST: aspartate aminotransferase
BCS: body condition score
BHB: beta-hydroxybutyrate
BUN: blood urea nitrogen
CAT: catalase
COR: cortisol
CRE: creatinine
DHA: docosahexaenoic acid
DIM: days in milk
DMI: dry matter intake
DPA: docosapentaenoic acid
EC: electrical conductivity
ECM: energy-corrected milk
EPA: eicosapentaenoic acid
FA: fatty acids
FCM: fat-corrected milk
FE: feed efficiency
GCG: glucagon
GH: growth hormone
GLB: globulin
GLU: glucose
GSH-PX: glutathione peroxidase
HDL-C: high-density lipoprotein cholesterol
HMDB: Human Metabolome Database
HPT: haptoglobin
IgA: immunoglobulin A
IgG: immunoglobulin G
IGF-1: insulin-like growth factor 1
IL-1β: interleukin-1β
IL-6: interleukin-6
IL-10: interleukin-10
INS: insulin
LDH: lactate dehydrogenase
LDL-C: low-density lipoprotein cholesterol
LH: long-lived high-yielding cows
LL: long-lived low-yielding cows
LPE: lysophosphatidylethanolamine
LPS: lipopolysaccharide
MD: milk density
MDA: malondialdehyde
MF: milk fat
MFP: milk freezing point
MFY: milk fat yield
ML: milk lactose
MP: milk protein
MPY: milk protein yield
MSNF: milk solid-not-fat
MTS: milk total solids
MUFA: monounsaturated fatty acids
MY: milk yield
NEFA: non-esterified fatty acids
PICRUSt2: Phylogenetic Investigation of Communities by Reconstruction of Unobserved States;
PRL: prolactin
ProtC: Protein carbonyl
PS: phosphatidylserine
PUFA: polyunsaturated fatty acids
RpH: rumen Ph
SAA: serum amyloid A
SOD: superoxide dismutase
T3: triiodothyronine
T4: thyroxine
T-AOC: total antioxidant capacity
TBA: total bile acids
TC: total cholesterol
TG: triglyceride
T-GSH: total glutathione
TNF-*α*: tumor necrosis factor-α
Total VFA: total volatile fatty acid
TP: total protein
*γ*-GGT: γ-glutamyl transferase
