# Supplementary material for: Comparative study of milk microbiota and metabolome in long-lived dairy cows with different persistent production capacities
Source: Front Microbiol. 2026 Jan 6;16:1725031. doi: 10.3389/fmicb.2025.1725031 (PMC12816256; doi:10.3389/fmicb.2025.1725031)
Supplement: Supplementary file 1 [file Table_1.DOCX]

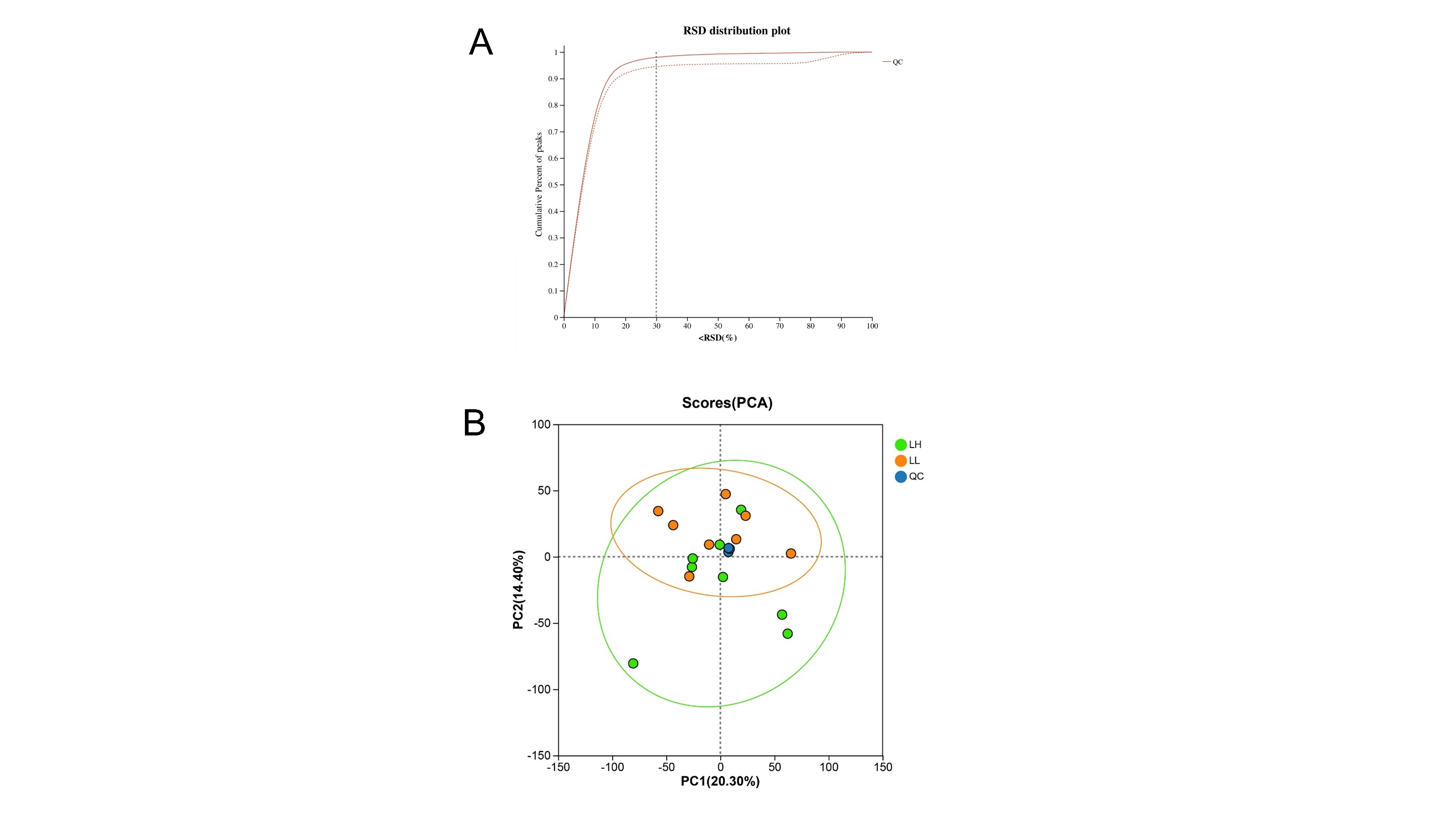


Figure S1. QC evaluation of metabolomics data. (A) Cumulative distribution of RSD values for metabolic features in QC samples (solid line) and experimental samples (dashed line). The dashed line indicates the 30% RSD quality-control threshold. (B) PCA showing clustering patterns of QC and experimental samples. QC = quality control; RSD = relative standard deviation; PCA = principal component analysis.
