# Supplementary material for: Comparative study of milk microbiota and metabolome in long-lived dairy cows with different persistent production capacities
Source: Front Microbiol. 2026 Jan 6;16:1725031. doi: 10.3389/fmicb.2025.1725031 (PMC12816256; doi:10.3389/fmicb.2025.1725031)
Supplement: Supplementary file 2 [file Table_2.DOCX]

| **Table S1. Ingredients of the basal diet (%, DM basis).** | |
| --- | --- |
| Item | Content |
| Ingredients, % |  |
| Alfalfa | 4.31 |
| Corn silage | 51.26 |
| Whole cottonseed | 2.46 |
| Corn grain, ground | 6.56 |
| Flaked corn | 4.92 |
| Beet pellets | 1.23 |
| Soybean meal | 7.38 |
| Extruded soybeans | 1.64 |
| Corn gluten meal CP60% | 0.51 |
| Rapeseed meal | 0.88 |
| DDGS | 2.05 |
| Calcium fatty acids | 0.62 |
| Sodium bica rbonate | 0.62 |
| Urea | 0.14 |
| Water | 14.35 |
| Premix^1^ (lactation period) | 1.07 |
| Nutrition composition, % |  |
| Dry matter | 55.80 |
| Crude protein | 16.35 |
| Starch | 18.50 |
| Neutral detergent fiber | 32.10 |
| Acid detergent fiber | 18.45 |
| Calcium | 1.00 |
| Phosphorus | 0.40 |
| NE_L_, MJ/kg^2^ | 7.14 |
| DM = dry matter; CP = crude protein; DDGS = distillers dried grains with solubles.  ^1^Premix2 (lactation period): Each kilogram of premix dry matter contains vitamin A 134.57 kIU, vitamin D 36.77 kIU, vitamin E 825.85 IU, Fe 985.97 mg, Cu 183.77 mg, Zn 919.99 mg, Mn 915.14 mg, Se 7.36 mg, and Co 13.80 mg.  ^2^Net energy for lactation was calculated according to National Academies of Sciences et al. (2021). | |

References

National Academies of Sciences, E., Medicine, Division on, E., Life, S., Board on, A., Natural, R., et al. (2021). in *Nutrient Requirements of Dairy Cattle: Eighth Revised Edition*. (Washington (DC): National Academies Press (US) Copyright 2021 by the National Academy of Sciences. All rights reserved.).
