# Supplementary material for: Comparative study of milk microbiota and metabolome in long-lived dairy cows with different persistent production capacities
Source: Front Microbiol. 2026 Jan 6;16:1725031. doi: 10.3389/fmicb.2025.1725031 (PMC12816256; doi:10.3389/fmicb.2025.1725031)
Supplement: Supplementary file 3 [file Table_3.DOCX]

**Table S2 Sequencing depth and rarefied numbers for each sample.**

| Sample | Sequence number | Rarefied number |
| --- | --- | --- |
| LH1  LH2  LH3  LH4  LH5  LH6  LH7  LH8 | 41596  50782  45510  57864  57295  46424  58149  59914 | 20000  20000  20000  20000  20000  20000  20000  20000 |
| LL1  LL2  LL3  LL4  LL5  LL6  LL7  LL8 | 48844  55032  43546  56272  47392  58423  51145  70664 | 20000  20000  20000  20000  20000  20000  20000  20000 |
| Average  SD | 60945  7100 | 20000  0 |

SD =standard deviation.
